# Acute respiratory viral diseases in Amazonas, Brazil: epidemiological patterns and implications for health surveillance

**DOI:** 10.1590/0037-8682-0102-2026

**Published:** 2026-08-07

**Authors:** Rahyja Teixeira, Felipe Nery, Erica Carvalho, Ivana Andrade Vieira Neves, Fernando Val, Monique Freire, Felipe Murta, Maria Paula Gomes Mourão, Wuelton Monteiro, Vanderson Sampaio

**Affiliations:** 1 Escola de Ciências da Saúde, Universidade do Estado do Amazonas, Manaus, AM, Brasil.; 2 Departamento de Ensino e Pesquisa, Fundação de Medicina Tropical Dr. Heitor Vieira Dourado, Manaus, AM, Brasil.; 3 Instituto de Ciências Biológicas, Universidade Federal do Amazonas, Manaus, AM, Brasil.; 4 Departamento de Patologia e Medicina Legal, Universidade Federal do Amazonas, Manaus, AM, Brasil.; 5 Duke Global Health Institute, Duke University, Durham, USA.; 6 Instituto Todos pela Saúde, São Paulo, SP, Brasil.

**Keywords:** Severe acute respiratory syndrome, Influenza-like illness, SARS-CoV-2, Spatial epidemiology

## Abstract

**Background:**

Acute respiratory viral diseases are a major public health challenge in the Brazilian Amazon, where ecological, logistical, and social factors shape patterns of transmission and response.

**Methods:**

This study aimed to analyze the epidemiological patterns and temporal-spatial distribution of Influenza-like illness (ILI) and severe acute respiratory syndrome (SARS) in the state of Amazonas, Brazil, between 2015 and 2025, distinguishing SARS-CoV-2 and non-SARS-CoV-2 etiologies using data from OpenDataSUS.

**Results:**

Incidence peaks occurred in early 2021 and 2022, with pronounced regional disparities. The highest burdens were concentrated in specific municipalities, with Manaus exhibiting an intermediate incidence and playing a central role in case notifications and healthcare provision.

**Discussion:**

We describe the integration of surveillance systems, laboratory networks, and healthcare infrastructure, which enabled improvements in diagnosis, monitoring, and care. The region’s response model, centered in Manaus, includes primary-to-tertiary care coordination, molecular diagnostics, telemedicine, and mobile health units for Indigenous and remote areas. Research efforts during the COVID-19 pandemic provided critical insights into therapeutic strategies, immunopathology, and long-term sequelae, while also highlighting persistent inequities and diagnostic gaps. Our findings underscore the co-circulation of multiple respiratory pathogens and the need for continued genomic and syndromic surveillance. Future strategies must address regional disparities, support decentralized diagnostics, and expand clinical research. Strengthening integrated health systems in the Brazilian Amazon is essential for timely, equitable responses to emerging respiratory threats.

## INTRODUCTION

Acute respiratory viral diseases are a major public health concern in the Brazilian Amazon, where unique ecological, social, and logistical factors shape patterns of transmission, surveillance, and care. Respiratory infections remain important causes of morbidity and hospitalization, particularly among socially vulnerable populations and residents of remote areas^1^ ^,^ ^2^.

Acute respiratory viral diseases are major contributors to global morbidity and mortality^3^. In Brazil, surveillance of influenza-like illness (ILI) and severe acute respiratory syndrome (SARS) has progressively expanded since the influenza A(H1N1)pdm09 pandemic and was substantially strengthened during the COVID-19 pandemic through systems such as SIVEP-Gripe and e-SUS Notifica. Recent evaluations of the Brazilian acute respiratory infection surveillance system demonstrated important regional inequalities in reporting completeness and timeliness, particularly in the North region, where logistical and structural barriers may compromise epidemiological monitoring and laboratory investigation^4^ ^,^ ^5^. These findings are especially relevant for the Amazon region, where geographic dispersion, limited healthcare access, and territorial heterogeneity influence both disease transmission dynamics and surveillance capacity^6^ ^,^ ^7^.

The state of Amazonas presents additional challenges for respiratory disease surveillance due to its vast geographic dispersion, limited health infrastructure in remote municipalities, dependence on river transportation, and high levels of social vulnerability^8^ ^,^ ^9^. These factors may directly affect healthcare access, diagnostic capacity, and timely case notification, particularly in rural and Indigenous territories^10^. 

At the same time, previous studies have shown that respiratory virus circulation in northern Brazil differs from patterns observed in other regions of the country, with influenza activity frequently concentrated in the first months of the year^11^. This seasonal pattern has important implications for surveillance and vaccination strategies in the Amazon region.

Despite advances in respiratory surveillance and increasing availability of public health data, comprehensive longitudinal analyses integrating temporal, spatial, and epidemiological patterns of respiratory syndromes in Amazonas remain limited. Most available studies focus on specific pathogens or restricted time periods, limiting a broader understanding of respiratory disease dynamics in the region.

In this work, we present a comprehensive epidemiological analysis of acute respiratory diseases in Amazonas, covering the temporal and spatial evolution of ILI and severe acute respiratory syndrome (SARS) from 2015 to 2025. Using official data from OpenDataSUS, we characterize incidence trends, highlight key moments of viral circulation, and assess the distribution of disease burden across municipalities. We also examine the structure of the healthcare network, the evolution of scientific evidence in the region, and future directions for respiratory disease surveillance and control in the Amazonian context.

## METHODS

The data analyzed in this study were obtained from OpenDataSUS, the official open-data platform of the Brazilian Unified Health System (Sistema Único de Saúde, SUS), managed by the Department of Information and Informatics of SUS (DATASUS/ dados.gov.br ). The inclusion criteria comprised confirmed SARS cases registered in the Influenza Epidemiological Surveillance Information System (SIVEP-Gripe) between 2015 and 2025, identified according to the final classification recorded in the surveillance system and following laboratory, clinical-epidemiological, clinical, and clinical-imaging criteria established by the Brazilian Ministry of Health, as well as confirmed records of ILI reported in the e-SUS Notifica system in the state of Amazonas between 2020 and 2025.

Preliminarily, the databases were assessed for data quality, particularly regarding completeness. Records with less than 70% completeness were excluded from the analysis. Duplicate records and inconsistencies were also identified and excluded after evaluation of the data quality findings.

The data processing phase was carried out using the R software (v4.5.1)^12^ and the RStudio development environment (v2025.05.1+513)^13^, including correction, cleaning, processing and integration of the databases. For these steps, specific packages were used to optimize this process, such as "*tidyverse*", "*patchwork*", "*data.table*", and "*geobr*", ensuring greater efficiency in the manipulation of the data sets. The final version of the dataset was used to generate analytical graphs and a thematic map in the QGIS software, allowing the spatial visualization of the results.

The graphs were generated from frequency tables grouped by month and year of notification, representing the temporal evolution of ILI and SARS cases in the state of Amazonas. In the SARS database, records were classified as SARS-CoV-2 or non-SARS-CoV-2 based on the available etiological information. For the ILI database, only confirmed Influenza-like illness cases were included. After grouping, we incorporated data on the estimated average population of each municipality, obtained from the Brazilian Institute of Geography and Statistics (IBGE), using a five-year average for ILI and a ten-year average for SARS. Based on these estimates, we calculated the incidence per 100,000 inhabitants to standardize the rates and enable comparisons across different periods and municipalities. These incidence values were also used to generate thematic maps. Geographic coordinates corresponding to the centroid of each municipality were added, enabling spatial mapping and the creation of a consolidated georeferenced dataset. This dataset was exported to QGIS, where kernel density maps were produced to represent the spatial distribution of incidences across the state of Amazonas.

As the study used only aggregated public-domain data with no individual identifiers, it was exempt from ethical review under Conselho Nacional de Saúde (CNS) Resolution No. 510/2016.

## RESULTS

**FIGURE 1:**
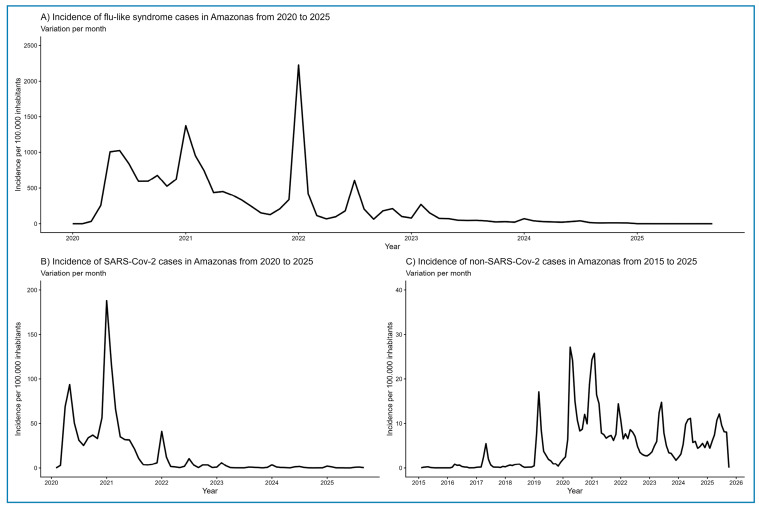
The incidence of SARS cases associated with SARS-CoV-2 remained lower than that of flu-like syndrome cases but exhibited a similar pattern of monthly variation, with sharp peaks in January and February 2021 (188.12 and 117.52, respectively): **(A)** Incidence Influenza-like illness cases in Amazonas from 2020 to 2025; **(B)** Incidence SARS-Cov-2 cases in Amazonas from 2020 to 2025; **(C)** Incidence of non-SARS-Cov-2 cases in Amazonas from 2015 to 2025.

The incidence of ILI cases exhibited pronounced fluctuations between 2020 and 2023, followed by a stabilization trend from 2024 onward. The highest values were recorded in January 2022 (2,225.2), followed by January 2021 (1,376.34), corresponding to periods of intense viral circulation and increased strain on healthcare services. The lowest incidence was observed in September 2025, at 0.02 (Figure 1A).

The incidence of SARS cases associated with SARS-CoV-2 and those not associated showed a similar temporal pattern between 2020 and 2022, with sharp peaks in the early months of each year (Figure 1A and 1B ). The highest incidence of SARS-CoV-2 cases was recorded in January and February 2021, corresponding to the peak of the pandemic in the state. Among the other etiological classifications, the "unspecified SARS" category showed the highest incidence, especially between 2020 and 2021, when limited diagnostic capacity prevented identification of the causative agent. The highest incidence in this category was observed in April 2020 (27.13), followed by February 2021 (24.59), while the lowest was recorded in February and August 2018 (0.02). 

SARS cases not associated with SARS-CoV-2 showed the lowest incidence rates among the three groups analyzed, while maintaining a comparable temporal pattern, particularly between 2019 and 2023. The highest rates were recorded in April 2020 (27.18), followed by February 2021 (25.82), whereas the lowest rates occurred in August and September 2015 (0.03). This indicates that, despite differences in magnitude, all categories followed a similar seasonal trend, characterized by increased incidence in the early months of each year (Figure 1C).

**FIGURE 2:**
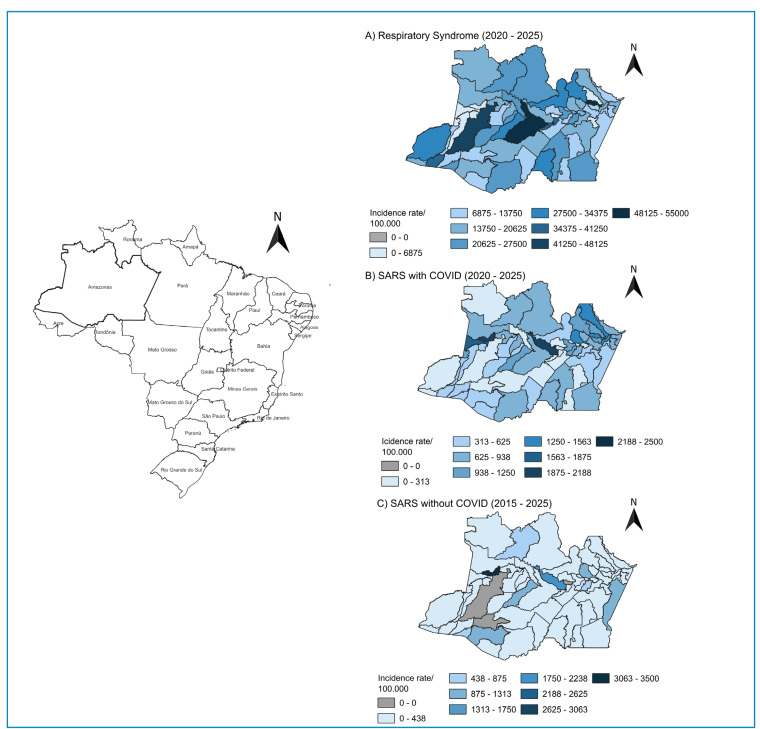
Spatial distribution of incidence rates across municipalities in the state of Amazonas, Brazil, from 2015 to 2025: **(A)** Influenza-like illness; **(B)** severe acute respiratory syndrome (SARS) associated with SARS-CoV-2; and **(C)** SARS not associated with SARS-CoV-2.

Between 2020 and 2025, the spatial distribution of the incidence of ILI e cases in the state of Amazonas revealed pronounced regional disparities. The highest values were recorded in the municipalities of Coari (53,692.95), Itapiranga (51,143.66), and Jutaí (44,887.31), while the lowest were observed in São Sebastião do Uatumã (1,347.91), Santo Antônio do Içá (2,113.88), and Anamã (3,141.67). Manaus presented an intermediate incidence of 16,181.30, lower than in the highest-risk municipalities but above the state average (Figure 2A).

The incidence of severe acute respiratory syndrome (SARS) associated with SARS-CoV-2 showed a heterogeneous spatial pattern across municipalities. The highest rates were observed in Codajás (2,133.30), Tonantins (1,998.00), and Santo Antônio do Içá (1,566.20), indicating areas of intense viral circulation. The lowest rates were observed in Anamã (96.95). Manaus had an incidence of 1,341.49, ranking among the municipalities with the highest burden (Figure 2B). In contrast, the lowest values were recorded in Itamarati (52.78) and Careiro da Várzea (58.53) (Figure 2B). For SARS cases unrelated to SARS-CoV-2 infection, the spatial distribution was more concentrated. Tonantins (3,356.90), Codajás (2,184.42), and Tefé (1,247.68) had the highest incidence rates during the study period. No cases were reported in Anamã, Itamarati, or Jutaí, resulting in zero incidence in these municipalities. Among the areas with low incidence, Novo Aripuanã (4.13), Beruri (4.91), and Urucará (5.22) were notable. Manaus showed an intermediate incidence of 977.41, below the highest-risk areas but still representing a significant site of case notifications (Figure 2C). These estimates should be interpreted considering differences in municipal population size, as incidence rates may be amplified in municipalities with smaller population denominators.

**FIGURE 3:**
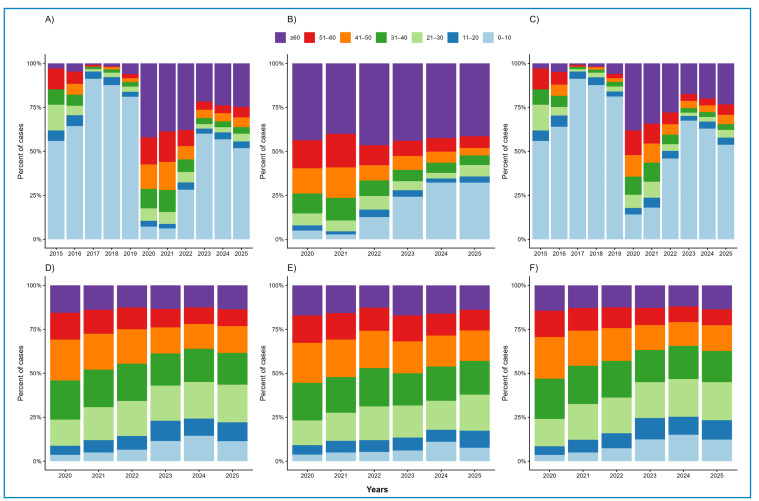
Age-stratified distribution of respiratory syndromes from 2015 to 2025. **(A)** Severe Acute Respiratory Syndrome (SARS), all etiologies combined; **(B)** SARS cases attributed to COVID-19; **(C)** SARS cases excluding COVID-19; **(D)** ILI, all etiologies combined; **(E)** FS cases attributed to COVID-19; and **(F)** ILI cases excluding COVID-19. The figure presents the annual proportional distribution of cases according to age group, allowing visual comparison of age-specific patterns across respiratory syndrome categories and study years.

Between 2015 and 2019, SARS cases were predominantly observed in children aged 0-10 years. During the COVID-19 pandemic period (2020-2022), the age distribution changed markedly, with older adults (≥60 years) representing the largest proportion of reported cases. COVID-19-specific analyses also demonstrated a predominance of older age groups across the study period. In contrast, influenza-like illness cases were distributed across multiple age groups, with adults accounting for a substantial proportion of notifications and children aged 0-10 years representing a smaller share of cases (Figure 3).

## DISCUSSION

This study provides a ten-year epidemiological overview of influenza-like illness and severe acute respiratory syndrome in Amazonas, demonstrating marked temporal variability, heterogeneous geographic distribution, and differences across age groups and etiological profiles. The integration of syndromic, laboratory, genomic, and metagenomic surveillance data allowed a broad characterization of respiratory disease dynamics in the region.

The findings highlight the complex epidemiological dynamics of respiratory syndromes in Amazonas, characterized by marked geographic heterogeneity, age-specific patterns, and distinct seasonal behavior. The concentration of influenza activity during the first months of the year reinforces previous evidence that respiratory virus seasonality in the Amazon differs from patterns observed in southern Brazil, supporting the need for regionally tailored surveillance and vaccination strategies^14^ ^,^ ^15^. Furthermore, the post-pandemic predominance of RSV among infants suggests a shift in respiratory pathogen circulation that may increase the burden on pediatric healthcare services^16^ ^-^ ^18^. The observed age distribution of severe respiratory disease also reflects the substantial impact of SARS-CoV-2 on regional epidemiology, particularly among older adults and vulnerable populations^19^ ^-^ ^21^. Together, these findings underscore the importance of maintaining integrated respiratory surveillance systems capable of detecting changes in pathogen circulation and supporting timely public health responses.

The COVID-19 pandemic substantially altered respiratory surveillance dynamics during the study period. Non-pharmaceutical interventions, changes in healthcare utilization, expanded molecular testing capacity, and shifts in surveillance priorities may have influenced the circulation and detection of other respiratory viruses^22^ ^-^ ^24^.

Marked heterogeneity among municipalities likely reflects differences in healthcare access, laboratory infrastructure, and surveillance sensitivity across Amazonas. In remote municipalities, reduced diagnostic capacity and logistical challenges may contribute to underdetection and underreporting of respiratory syndromes. Recognizing these challenges as being systemic underscores the need for strengthened logistical support and expanded decentralization of diagnostic services to improve surveillance sensitivity and equity across the Amazon region^25^ ^,^ ^26^. 

This study has important limitations that should be considered when interpreting the findings. First, the analyses are primarily descriptive and were not designed to establish causal relationships between respiratory virus circulation and demographic, climatic, socioeconomic, or healthcare-related factors. Therefore, interpretations regarding potential drivers of viral spread should be considered contextual and hypothesis-generating rather than inferential conclusions. The study relied on secondary surveillance data obtained from heterogeneous reporting systems, which are subject to underreporting, delays in notification, inconsistencies in data completeness, and variations in diagnostic capacity across municipalities. These limitations are particularly relevant in remote areas of Amazonas, where access to healthcare services and laboratory infrastructure remains uneven. Consequently, the apparent absence or lower incidence of cases in some municipalities may reflect surveillance limitations rather than true epidemiological absence. The COVID-19 pandemic likely influenced healthcare-seeking behavior, testing priorities, notification practices, and circulation dynamics of other respiratory viruses, potentially affecting temporal comparisons throughout the study period. Additionally, changes in surveillance protocols and diagnostic availability over time may have contributed to fluctuations in reported case numbers^27^ ^-^ ^30^.

Despite these challenges, the integration of epidemiological, laboratory, genomic, and metagenomic surveillance data represents an important strength of this study and highlights the potential of combined surveillance strategies for monitoring respiratory pathogens in geographically complex regions. Combining genomic and metagenomic data with clinical and epidemiological information can enhance the sensitivity and specificity of surveillance systems, allowing for real-time risk assessment and more effective outbreak containment strategies, especially in biodiverse settings with high zoonotic risk^31^ ^,^ ^32^.

Finally, the study did not employ advanced spatial, temporal, or spatio-temporal modeling approaches capable of formally evaluating associations between epidemiological patterns and environmental or structural determinants. Future studies using inferential analytical frameworks may further clarify these relationships and expand understanding of respiratory virus dynamics in the Amazon region.

In conclusion, this study provides a comprehensive descriptive overview of respiratory syndromes in Amazonas over a ten-year period and reinforces the importance of strengthening integrated respiratory surveillance systems in the Brazilian Amazon. Continued investment in laboratory capacity, surveillance coverage, and longitudinal monitoring will be essential to improve preparedness for seasonal and emerging respiratory pathogens.

## Data Availability

Research data is only available upon request.
